# On mental pain and suicide risk in modern psychiatry

**DOI:** 10.1186/s12991-024-00490-5

**Published:** 2024-01-16

**Authors:** Maurizio Pompili

**Affiliations:** Department of Neurosciences, Mental Health and Sensory Organs, Suicide Prevention Center, Sant’Andrea Hospital, Sapienza University of Rome, Rome, Italy

**Keywords:** Suicide risk, Mental pain, Empathy, Phenomenology, Psychiatry

## Abstract

Facing suicide risk is probably the most difficult task for clinicians when dealing with patients in crisis. It requires professional, intellectual, and emotional efforts. Suicide risk assessment can sometimes be distressing for clinicians, and such a state may favour the avoidance of an in-depth exploration of suicidal thoughts and behaviour. Patients often feel subjected to interpersonal assessments with little opportunity to explore their perspectives. The "One size fits all" approach tends to create distance and paradoxically contributes to an increase in the risk of suicide. Traditional clinical factors may be of limited value if a shared understanding of the patient’s suicide risk is missed. To understand the suicidal mind, it is necessary to take the point of view of the subject in crisis. In this essay, the “operational model of mental pain as a main ingredient of suicide” provided by Edwin Shneidman is overviewed with the aim of a better empathic understanding of patients’ sufferance. With a phenomenological approach, the suicidal crisis appears as a complex, pervasive state rather than as a symptom of a mental disorder, as the new paradigm also suggests. In this regard, the "mentalistic" aspects of suicide propose a broader insight into the suicidal scenario far beyond the diagnosis of psychiatric disorders. In this article, the perspective of individuals who deem their mental pain to be intolerable is described to make sense of their ambivalence between the wish to die and the wish to live that can prevail if relief is provided.

## Introduction

To shed light on the suicidal mind and, therefore, to adjunct emphasis on the need for an empathic understanding of the suffering of the individual in crisis, this essay is provided. I aim to recollect some of the basic and original elements on which suicide risk is related to mental pain and how to make sense of it, using concepts elaborated by Edwin Shneidman, considered the father of suicidology, who, during the last years of his life gave me insightful directions in suicidology as a mentor.

It is now clear that in the suicidal risk, great help is referred to listening and sharing the experience of suffering. Otherwise, patients often feel subjected to interpersonal assessments with little opportunity to explore their perspectives. The "standard and for all" approach, such as paying attention to generic risk factors, tends to create distance and paradoxically contributes to an increase in the risk of suicide. An extensive meta-analysis of the current body of literature on the topic resulted in the discovery that risk factors had limited usefulness and imprecision in predicting suicidal behavior. According to the authors, the effectiveness of risk variables in predicting outcomes has not improved in the past 50 years and has remained modest even in more frequent follow-up periods than usual [1].

To adequately set the scene, let me begin by saying that, in psychiatry, empathically understanding others is a quintessential pillar. One may wonder if the suicidal phenomenon is fully explained with endless contributions but with far less understanding as a private human condition. Although not necessarily overlapping concepts, contents of the psychopathological perspective and items of the suicidal spectrum may take advantage of seminal contributions in psychiatry and general psychopathology. Jaspers reminds us: (1) we sink ourselves into the psychic situation and *understand genetically by empathy* how one psychic event emerges from another; and (2) we find by repeated experience that a number of phenomena are regularly linked together, and on this basis, *we explain causally* [2]. The understanding vs. explaining dichotomy often impacts our clinical duties, so we can explain something without understanding it, because experience as inner reality is not possible. However, as for understanding, according to Jaspers “*in the natural sciences we find causal connections only but in psychology our bent for knowledge is satisfied with the comprehension of quite a different sort of connection. Psychic events 'emerge' out of each other in a way which we understand. Attacked people become angry and spring to the defence, cheated persons grow suspicious. The way in which such an emergence takes place is understood by us, our understanding is genetic. Thus, we understand psychic reactions to experience, we understand the development of passion, the growth of an error, the content of delusion and dream; we understand the effects of suggestion, an abnormal personality in its own context or the inner necessities of someone's life. Finally, we understand how the patient sees himself and how this mode of self-understanding becomes a factor in his psychic development.* [2]*.*

Objective symptoms can be clearly and convincingly demonstrated to anyone who is capable of perceiving through their senses and thinking logically. However, to comprehend subjective symptoms, they must be attributed to a process that is typically labeled as "subjective," distinguishing them from sensory experience and logical thinking. The sense organs are unable to directly perceive subjective symptoms, but they can be understood by immersing oneself, so to speak, in the other person's psyche; this is known as empathy. The spectator can only internalize these experiences by actively engaging in the other person's encounters, rather than relying on intellectual exertion [3].

Without understanding their meanings and structure, we cannot determine the necessary relationships between two facts through the objective gaze. Understanding, however, penetrates the psychic to grasp its origin within the psychic itself: understandable relationships are a sort of "causality from within", which can be grasped clearly and immediately, as happens when we understand the anger of a person attacked or the joy that comes from reciprocated love. Unlike causal rules, derived inductively from the cases in which they manifest themselves, comprehensible relations are not subordinated to external facts and their frequency but arise from the evidence of the structural link between phenomena. Therefore, any understanding of psychic phenomena cannot be presented as knowledge and is not neutral: it does not limit itself to saying "what is", but always implies a form of subjective participation. In other words, there are processes that we can understand as they are close to our internal experience. At the same time, others are foreign to our ability to understand them, as they cannot be deciphered based on internal experiences. In this discussion, it is possible to explain something without understanding it. Much of the emphasis on suicide risk is in explaining rather than understanding it.

Although important in many regards, in this specific discussion, rather than sustaining the Kraepelinian paradigm, which saw every mental illness as a disease of the brain, the innovation that brought about this line of thinking points to the doctor/patient relationship based on patients' experiences into consideration not only as symptoms but as expressions of their inner world.

​To comprehend suicide, it is crucial to incorporate into new paradigms the first-person information derived from personal experiences as well as the methodologies used to capture and evaluate this data. These subjective perspectives and approaches are necessary additions to objective facts and conventional scientific procedures. We need to bridge this “explanatory gap” for a complete understanding of suicide risk. Apart from the third person, objective knowledge of suicide, which is now well-represented, clinicians should also be familiar with first-person, subjective experience and ask themselves, “What’s it like to be suicidal?” to fill in the “epistemological gap” for a more complete understanding of suicide.

As suicide is a major public health problem worldwide, clinicians are now required to embrace new paradigms for a proper understanding of the suicidal scenario [4].

Unlocking the suicidal mind is one the most challenging of all tasks. Many models describing suicide fail to fully understand this multifaceted human condition properly. Over time, man has sought different ways to make sense of suicide. To date, it is believed that a multidisciplinary approach can explain the complexity of an event by not reducing it to superficial or partial visions that lead to the removal of all elements that make up the tragedy but also the intrinsic emotional truth of every single suicidal event. We are also reminded that our major difficulty is figuring out how the suicidal wishes emerge and how the suicidal subject experiences such an event. We need to understand the thoughts and feelings of those who live the suicidal process.

Among the various approaches to explaining the topic of suicide, there are the philosophical and theological approach; the literary approach derived from the study of personal documents; the demographic approach; the sociological approach; the sociocultural approach; the interpersonal approach; the psychodynamic approach; the biological approach; the psychological approach; and the medico-psychiatric approach [5]. The latter emphasizes that suicide is mostly a result of a psychiatric disorder or medical condition and is a direct outcome of the morbid state. According to this premise, suicide is often classified as a symptom. The literature, mostly from psychological autopsy studies, emphasises that 90% of suicidal subjects had a mental disorder; in this way, it suggests that if one had intervened in the disorder, the lethal outcome could have been prevented in some cases. Such a conclusion is now challenged by scholars who see a too narrow view for a comprehensive phenomenological understanding of suicide [6, 7].

Psychological autopsies, as reported in the literature, involve some of the limits of such procedures, pointing to a lack of standardised instruments for assessing psychopathology, life events, and the procedure for interviewing informants. Studies investigated concordance between different informants (caregivers, spouses, clinicians, etc.) on the diagnoses of suicides and showed that agreement ranges from poor to moderately good. The informant’s profile might significantly influence the information obtained on cases, with an overlap in data from different sources that is far from perfect. It must also be said that most psychiatric patients do not die by suicide, so some clarifications are required.

It has been evident in recent decades that attempting to evaluate and handle the risk of suicide without recognizing and acknowledging a patient's distress would be counterproductive for several reasons. [8]. Suicide ideation or suicide risk, in general, should not be at the same level as symptoms of psychiatric disorders, for instance, in the case of depression. Instead, depression may be a risk factor for suicide. Still, the complete understanding of the suicidal scenario lies on a complex dimension, where psychiatric disorders are important contributors, although not exclusive, to suicide risk.

Fava et al. [9] stressed the role of transdiagnostic features of mental pain as it is associated with various psychiatric disorders and pointed to the DSM-5 characterisation of mental pain as “clinically significant distress” caused by the symptoms of a psychiatric disorder. These authors broadly overviewed features of mental pain in patients suffering from psychiatric disorders and traumatic events. In such a view, mental pain points to patient-reported outcomes, that is, any report coming directly from patients about how they function or feel concerning a health condition or its therapy. These authors described mental pain in depression as a uniquely aversive, anguished, or uncomfortable experience that is characterised by painful tension and torment.

While perspectives from Shneidman point to a more psychological dimension related to thwarted psychological needs, in such a conceptualisation, a closer view of psychiatric-oriented phenomenological understanding of mental disorders is provided, but regardless of how mental pain is conceptualised in its origins, it constitutes a strategic construct for the better characterisation of psychiatric patients and their therapeutic plans.

Michel [6] recently stressed that suicide and attempted suicide as human phenomena do not fit into this traditional medical model, which may prevent individuals in crisis from seeking help. There is the risk of ignoring an individual’s private personal experience; they feel that the busy medical professional, trained to provide a diagnosis, will not understand their inner struggles, their loss of self-respect, and their self-hate. Schechter and Maltsberger [10] underline that the exclusively medical approach, aimed at detecting symptoms (assuming it is correct to use this term), risks disappointing all of the patient's expectations, which refer to primary needs, such as being understood, accepted, and welcomed, etc. Therefore, there is even a possibility of leaving the patient with an even greater feeling of loneliness.

When investigating sadness as a human experience resulting from an array of unfavorable circumstances, Horwitz and Wakefield [11] pointed out that the DSM (for the edition at the time of their work) is not consistent when applying the definition of mental disorder to the diagnostic criteria established for specific disorders. Its criteria specified the symptoms that had to be present to justify a diagnosis. Still, ignoring any reference to the context in which the symptoms developed leads us to characterise normal responses to stressors as disturbing symptoms as part of a psychiatric disorder. The focus should be beyond the definition of a diagnosis and instead characterise individuals using psychopathological dimensions [12].

We must acknowledge the fact that the strength of this model lies in having traced the management of suicide risk to pharmacological therapies that are capable of making a difference in acute and chronic risk cases, which are not otherwise managed only with "psychotherapeutic" methods. The treatment of a symptomatic picture—anxiety, insomnia, agitation, lack of motivation, and dysphoria—which can represent the target of pharmacological therapies that the psychiatrist manages, is a fundamental part of the relief for reducing suicide risk. In addition, the circumstantiality of the event in the uniqueness and unrepeatability of the psycho-socio-cultural circumstances of time, places, and people that characterize the single event is extremely important. It implements measures with different priorities concerning the clinical case in the medical, psychological, psychiatric, social, cultural, welfare, economic, legislative, and political fields focused on a multi-determinate background [13]. In a new era for psychiatry brought about by new paradigms and proposals, such as the one in DSM-5, it seems reasonable to once more acknowledge the drama occurring in the mind of suicidal individuals; fine-tuning into this sufferance is a major goal for clinicians along with hopefully finding other solutions for such pain besides suicide [14]. Maris et al. [15, p. 28] stated that “*while suicidologists give lip service to the multidisciplinary study of suicide, in actual fact most of us have very narrow and specialised domain assumption—usually those related to our professional training and sub-disciplinary paradigms*”. In traditional suicidology, it is commonly accepted that individuals who are suicidal are enduring intense mental anguish or distress and that suicide may be seen as a means to alleviate this suffering to some extent. Despite enormous efforts over the past decades for the implementation of suicide prevention, encountering a suicidal individual remains a challenge for most professionals and the general public, while major institutions and educational entities often point to recognising warning signs. Unfortunately, such prodromic items are not always recognized or even lacking in suicidal individuals. We know that these individuals are ambivalent about ending their lives and, as far as we can explore the suicidal phenomenon, they wish to be saved; they want to live. Shneidman stated that *“thinking about the act [of suicide] ahead of time is a complicated, undecided, internal debate. Many black-or-white actions are taken on a barely pass vote”* [16]. Various structures describe the wish to die. Despite its simplicity, an exceptional model that highlights the significance of mental suffering in suicidal individuals has proven to be useful in understanding the suicidal mind, if only because of its clarity. The idea that a suicidal person endures excruciating psychological anguish or suffering and that, at least in part, suicide may be an attempt to end this suffering was originally put up by Edwin Shneidman [17]. He believed that the primary component of suicide is psychological distress. This paradigm emphasizes that suicide is not a journey towards death but rather an escape from intolerable feeling and unacceptably agonizing mental pain. According to this model, suicide is a way to end unbearable suffering. If tortured people could somehow cease their consciousness and yet survive, it would be their choice, which may explain why they experience negative feelings and an internal discussion that makes the flow of consciousness uncomfortable and ultimately leads them to the conclusion. When someone believes their psychological suffering is intolerable, they may die by suicide [18]. During the early phases of this process, suicide is considered an option, but it may be rejected several times. Shneidman [19] reported an emblematic process referring to the word ‘therefore’*: “almost every decision that a person makes (based on some unspoken reasoning in the mind): it is the logical bridge between almost every thought and every action (or deliberated inaction). Among all the...therefore, I...” sequences that are possible in the mind, one of the most important ones is contained in the words: ‘...therefore, I must kill myself”.*

As reported by Maris and colleagues [15, p. 29], the building blocks of a systematic theory of suicide include definition, basic concepts (lethality, motive, suicidal career, etc.), hypothesis, models, and research results. Regardless of such items, some concepts are so basic to suicide that they can be thought of as the commonalities of suicide.

I spent the past decades exploring such perspectives, especially investigating the role of mental pain as the main ingredient of suicide following Edwin Shneidman’s footsteps. He argued that the essential nature of suicide is psychological or mentalistic, meaning that each suicidal drama occurs in the mind of a unique individual [20].

He refers to mental pain as stating that psychological pain is the same as somatic or physical pain. “*It is how you feel as a person; how you feel in your mind or heart. It refers to how much you hurt as a human being. It is mental suffering; inner torment. It is called psychache (pronounced sik-ak). Psychache refers to hurt or misery. It is the pain of shame, or guilt, or grief, or humiliation, or hopelessness, or loneliness, or sadness, or anguish. It is how you feel inside. It is an ache in the mind*” [21]. Several articles addressed the role of mental pain as psychache to reconceptualise suicide risk in the realm of the intimacy of individuals’ negative emotions [20, 22–24].

The primary objective of this method is to emphasize that suicide risk arises from a sense of solitude and the experience of negative, distressing emotions. It recognizes suicide as an action taken to alleviate the terrible suffering of a tormenting existence. He was resolute in stating that each person has their own interpretation of what is considered "intolerable". He emphasized that challenges, pressures, or letdowns that may be manageable for one person could be insurmountable for another, depending on their mindset. To effectively anticipate and prevent suicide, it is imperative to comprehend the individual's interpretation of the term "intolerable." [16].

When the level of mental pain is deemed by that person to be unbearable, suicide is regarded as a potential choice to escape from such a condition. It would be oversimplifying to disregard any person contemplating suicide without a thorough comprehension of their anguish. According to this definition, suicide is not a symptom of a disease or a mental illness but rather follows a completely different recipe, which combines the idea that death or cessation can be a solution to the problem of seemingly unacceptably high levels of psychological suffering with extremely severe (unbearable) psychological pain [25]. On the other hand, diseases, mental illness, and other psychiatric symptoms may be risk factors for suicide. In a clinical situation, to bypass a direct confrontation with suicide risk, it appears wiser to ask key questions such “where do you hurt?” and “how may I help you?” It follows that given the role of the unbearable pain in energising suicide risk, the therapist’s main task is to mollify that pain. A way to achieve such a task is to understand the sources of psychological pain, such as shame, guilt, rage, loneliness and hopelessness, etc., as the theorisation of Shneidman supports the notion that sufferance stems from frustrated or thwarted psychological needs. These psychological needs that are specific for each individual include the need for achievement, affiliation, autonomy, counteraction, exhibition, nurturance, order and understanding.

## Understanding the suicidal mind

When trying to understand the suicidal mind, the main focus should be on what is behind the wish to die. Odd as it may be, death may not be a central topic, as most suicidal individuals wish to live but end up wanting to die as a solution to solve a state of mental suffering. Focusing on how to ameliorate mental pain may help to produce new patterns of action for wanting to live. Therefore, before considering the likelihood of dying by suicide, it seems relevant to pay attention to how perturbed the individual is in terms of inner turmoil, agitation and upset.

The subjects who experience the state of suffering, including restlessness, bewilderment, sadness and anxiety, which is often a precursor of suicide risk, try to contain this unpleasant state with behaviours that are ill-suited to the correct management of said negative emotions. The use of alcohol and drugs only temporarily allows the person not to think and to feel relief from agitation and anxiety. However, this comes at a very high price as it is a short-lived effect often followed by a worsening of mood, which then requires the further use of these substances without ever really getting rid of the primary problem. For many, it is the abuse of psychotropic drugs, primarily benzodiazepines, which can provide temporary relief but then impose dependence and habituation on the subject with the need to repeatedly increase the dosage, not to mention other effects that can be traced back to dysphoric and irritable states which are also often associated with the risk of suicide.

As mentioned, not thinking often becomes vital; not thinking means not having the thorns of pain made up of conclusions, elucubrations, inner dialogues, and infinite pessimistic, self-defeating and painful reasoning for which the subject cannot find solutions. This is how, for many, the end of the day is configured, as relief in thinking that maybe something will change. Sleep often brings comfort as thinking is stopped. Unfortunately, the night is often sleepless; the next day, the subject is even poorer in resources.

Since the disturbed state of the person experiencing a crisis motivates the person to consider suicide, knowledge of this situation is necessary to comprehend the suicidal mentality. Therefore, even though it seems obvious and straightforward, asking questions about the source of the pain and how it has intensified is a technique of intervention that those overseeing a person in crisis frequently overlook. Entering into the person's suffering in the internal conflict, which fundamentally involves ambivalence, allows one to stop such rumination and return the conversation to a place of life and hope. It seems reasonable to support the notion that *perturbation of the mind supplies the motivation for suicide; lethality, as the probability of dying through a specific method, is the fatal trigger*. However, Shneidman [26] stated “*admittedly, perturbation is difficult to define. In a sense, it encompasses all psychiatric nomenclature and terminology. But in the same way that we have established such concepts as “free-floating anxiety””*.

The state of suffering invites suicide as the only option left [22]. This results from an explosive mixture consisting of four constellations of emotional experience: heightened inimicality (acting against the individual’s best interest), the worsening of perturbation (refers to how disturbed the individual is; a state of being emotionally upset, disturbed, and disquieted, a state related to its dependency for action), increased constriction of intellectual focus and the narrowing of the mind’s content (dichotomous thinking). Fourth, the dea of cessation is the insight that it is possible to stop consciousness and put an end to suffering [27]. The realization that suffering may be ended and consciousness can be stopped is ultimately what the concept of cessation is all about. In this context, inimicality describes the mindset that causes a person to behave in an antagonistic way towards oneself, even to the extent of turning him into his own twisted adversary. Suicidal people experience this condition and struggle with a variety of issues, including their physical well-being, rejection, feelings of failure, pain, and other unpleasant emotions. Despite having resources at their disposal, the person cannot handle these problems. Family and friends may provide helpful assistance, yet the person cannot gain from them. The individual prioritizes their interpersonal experiences and positive recollections, which do not yield advantageous outcomes. To decipher the risk of suicide, clinicians must possess an in-depth knowledge of this intricacy. Under those circumstances, the person reaches the ultimate conclusion and, to cite Shneidman, “*the spark that ignites this potentially explosive mixture is the idea that one can put a stop to the pain. The idea of cessation provides the solution for the desperate person*” [26].

The notion of cessation arises when an individual contemplates the possibility of putting an end to the mental turmoil by means of death. The individual then realizes that death will resolve their experience by eradicating all those aspects that cause unacceptable suffering.

In addition to the main focus on psychological pain, Shneidman [25] also emphasized the concept of "press”. In this context, "psychological pressures" refers to the external factors that might cause stress or demand on an individual, sometimes even originating from within. These often encompass external factors, such as relational disputes, job loss, and large distressing life events. Presses are intricately connected to the sensation of being overwhelmed, which refers to the experience of being inundated by psychological demands.

The concept of "perturbation," defined by Shneidman [25], differs from mental pain, although not always easy to define. He claimed that perturbation encompasses being emotionally agitated, disrupted, and unsettled. According to Shneidman, perturbation refers to a state of cognitive restriction and a tendency to engage in self-harm or unwise actions. Perturbation refers to the patient's spontaneous inclination to take action to modify or amend their current intolerable circumstances. It is a fundamental psychological drive that serves as the primary motivator for all suicidal actions.

A tri-dimensional model encompassing mental pain, as described, is associated with a condition of the so-called “perturbation”; this is the upset of inner turmoil, including every diagnosis in the DSM and the press as conceptualised with pressures and vicissitudes of the outer world has been used to depict suicide as a result of the maximum level of sufferance in each of these three aspects.

The conventional classifications pertaining to suicide are somehow binary divisions, such as attempted, threatened, and completed. A more accurate perspective is to perceive them as potential continuums. Three continuous factors that are always present in the context of suicide are pain, disturbance, and pressure. The intensity of psychological pain can be measured on a scale ranging from hardly perceptible to extremely agonizing, using a numerical rating system from 1 to 5. Perturbation, can be assessed on a scale ranging from calm to highly distressing, using a rating system of 1 to 5.

Similarly, the external pressures and fluctuations of the outside world can also be evaluated on a scale of 1 to 5. A schematic cubic model for suicide can be derived from these thoughts. Suicide is said to happen when a person experiences a combination of intense suffering, disturbance, and pressure, referred to as the 5–5–5 cubelet. The therapeutic implication is to decrease at least one of those pertinent dimensions to a value of 4 or lower. Indeed, the most effective way to decrease the intense psychological pain that leads to suicide is first to decrease the intense disturbance that causes the pain. This can often be achieved by addressing the increased external pressure from strained interpersonal relationships, unemployment, school problems, and other factors.

According to Shneidman [25], *“the most direct way to reduce the heightened psychache (pain) that drives suicide is first to reduce the heightened perturbation that drives the pain—and frequently this can be done by addressing the heightened external press (of strained interpersonal relationships, unemployment, school problems, *etc*.)”.*

Of note is the fact that suicidologists have often referred to tunnel vision to describe a peculiar logic of suicidal individuals as a condition that derives from the state of suffering. Such a way of thinking postulates the increased constriction of intellectual focus; this refers to the narrowing of the mind’s content, with fewer options to cope with the suffering. In such a state, as part of tunnel vision, suicidal individuals may develop a dichotomous thinking process, because they reason with only two options when confronting the suffering that has become unbearable: wishing for either some specific (almost magical) total solution for their perturbation or cessation, in other words, suicide. The therapist must be vigilant for the patient's use of perilous suicidal language, such as the word "only" in phrases, such as "the only thing I can do" or "the only way to do it". They cannot see a way out, because the mind reacts to suffering with a logic restricting the possibility of finding a suitable solution to the pain-producing circumstances [22].

Such a restricted way of thinking comes after a long chain of option scans, with the rejection of the idea of suicide but ultimately accepting it as the best solution to the state of suffering. We acknowledge that the very core of tunnel vision is a gradual process guiding the individual into the only way out when other options fail. The individual convinces himself that, regardless of his efforts, suicide appears to be the only solution. This conclusion appears as the result of a peculiar logic of the suicidal mind, affected by overwhelming mental pain [22].

They can wander for hours, go away from home, or harbour a sense of great concentration made up of extremely intimate questions and answers on whether dying by suicide is right or wrong if it will be decisive or cause damage to those who remain.

Shneidman [27] outlined some doable strategies for assisting severely suicidal individuals and, as a result, saw suicide as a means of escaping agonizing mental suffering, with this suffering serving as the trigger for suicide. Shneidman [see [27], although described in various contributions of the author, such as [22]] identified several features that are found in at least 95% of individuals who die by suicide. He refers to these elements as "Commonalities of Suicide", which I will overview. Briefly, what follows is a list conveying such features which are almost always found in the suicidal mind that I aim to comment:

1) The common purpose of suicide is to seek a solution; it is never only an act without a conclusion. It pertains to the desire to escape a crisis or an intolerable condition that causes psychological distress. 2)The common goal of suicide is the cessation of consciousness. Indeed, suicide might be comprehended as an action that eradicates the individual's consciousness, where profound mental anguish resides, making it intolerable. Consequently, it is posited as the optimal resolution for the individual. 3) The common stimulus in suicide is intollerable psychological pain. If the individual desires to achieve cessation, they are attempting to escape psychological agony. Upon meticulous examination, suicides manifest as the convergence of a desire to stop the flow of concusses and the act of creating emotional distance due to excruciating mental anguish. When the degree of pain diminishes, suicide does not transpire. 4) The common stressor in suicide is frustrated psychological needs. Ironically, the individual contemplating suicide employs the act of suicide as a means to fulfil essential psychological needs that have been unmet. This, once again, leads to the inference that there could be numerous avoidable fatalities; 5) The common emotion in suicide is hopelessness–helplessness. Suicidal individuals often experience a sense of emotional despair and powerlessness. These individuals express a sense of hopelessness, believing that they have exhausted all options and that no one is capable of assisting in alleviating their suffering, to the extent that they contemplate suicide as the only viable solution. 6) The common cognitive state in suicide is ambivalence. Suicidal individuals commonly experience ambivalence in their cognitive state. Suicides are marked by a state of ambivalence, where individuals experience conflicting feelings towards life and death until they ultimately carry out the lethal act. Despite their preparations, they yearn for salvation from death. 7) The common perceptual state in suicide is constriction. Suicidal individuals exhibit a sense of temporary mental constriction that affects both emotions and intellect. Indeed, individuals contemplating suicide express sentiments such as "I had no alternative," "The sole path to demise was through exit," and "Taking my own life was the only viable option." This phenomenon is commonly referred to as tunnel vision, characterized by a limited range of choices and a mental focus on only two possibilities: a miraculous and joyful resolution or the act of ending one's life, known as suicide. In these instances, the principle of binary outcomes is enforced. 8) The common action in suicide is escape or egression. Suicides commonly occur as a means of escaping from difficult circumstances, an exodus from something distressing; 9) The common interpersonal act in suicide is communication of intention. Suicidal individuals typically communicate their intentions to others. From the initial psychological autopsies, it was discovered that in cases of uncertain deaths, which were ultimately categorized as suicides, there were indications of suicidal intention expressed in a more or less direct manner. These subjects engaged in psychotherapy intending to reduce mental distress in individuals who were at risk of suicide. Instead of expressing hostility, anger, depression, or withdrawal, they communicated their intention to commit suicide either verbally or through their behavior. In addition, the patterns of suicide observed were similar to the adaptive patterns of life exhibited by the individuals contemplating suicide. 10) The common pattern in suicide is consistent with life-long styles of coping. Suicide patterns are similar to adaptive patterns of life of the suicidal individual. In other words, by observing how a certain person has behaved in other difficult moments in their life, one can predict how the individual will approach the present crisis. Probably during other difficulties, that person has experienced the tendency to have dichotomous thinking and escape from pain. Although suicide, by definition, is an event never experienced. However, we can investigate the subjects' minds compared to lethal gestures by analyzing various kinds of mourning, separations, and losses.

As the flow of consciousness holds the thoughts referred to as negative emotions, and such thoughts are how the individual decides upon suicide, the cessation of such a process is the ultimate goal. In terms of emotional state, individuals in crises experience hopelessness and helplessness and, therefore, are trapped into conditions such as “there is nothing I can do (besides suicide) and there is no one who can help me (with the pain I am suffering)”. Notwithstanding the dreadful emotional situation, the individual in crisis feels the need to communicate their intention to die by suicide. Although not always traceable, such communications are provided directly or indirectly beforehand. It follows that there is a need to pay attention to any reference to suicidal wishes. Many people who die by suicide, even if ambivalent, consciously or unconsciously, leave clues about the intent, signs of unease, cries of impotence, or requests for intervention [22]. When experiencing a crisis, suicidal individuals often use adaptive schemes implemented in previous difficult moments of their lives. Therefore, a proper understanding of the history of each subject can shed light on the possible involvement of maladaptive solutions, such as, for example, the use of alcohol and drugs, as well as acting upon suicidal wishes.

Planning a suicide is often a drawn-out and challenging procedure. The individual starts to consider a good time; they need enough lead time to get ready. The person keeps having several conversations with themselves in the weeks and days leading up to the actual preparation and execution of the act. They may allude to their conviction that they are unworthy of anything, let alone others, that they have failed, and that they are a burden to their loved ones. This sets off an increasingly difficult task, during which there may also be a brief period of emotional elation in which the person begins to glorify suicide, sees it as a way out of a difficult situation, and arranges it as a plan to carry out without outside intervention. Consider doing something that, although it seems the wrong action to the subject, he feels is required to improve. Suicide is an act that is frequently planned out for a longer period than is commonly thought. The gesture does not turn into an impulsive one until beyond this point. The person who is in danger considers his loved ones during this time of planning the deadly act and feels regret for them. The person in question has considered ending their own life on multiple occasions; yet, each time it was considered, even though it was ultimately rejected, the option gained more significance. The person who is in danger of suicide starts to show signs at this point, indicating that they are sick of life, that they are thinking about dying, and that they would like to pass away. It is a human condition that can cause "emotional storms," significant ambivalent swings, and simultaneous adjustments to eating, sleeping, hygiene, and social interactions. The person who is in danger thinks about his loved ones during this time of planning the deadly deed and feels guilty and regret for coming up with such a horrible solution. In certain instances, complicated relationships with friends, family, or partners exist as well, to the point where the suicidal person nearly feels guilty for not getting enough support from them. The person in danger also feels alone in their emotional anguish and hopelessness. They also come to this conclusion after realizing they cannot express their pain to those tasked with assisting. Every person has a desire to die, and because every person has a unique set of motivations and thoughts, no two persons who are at risk of suicide are alike. According to Shneidman, the primary causes of psychological suffering are feelings of shame, guilt, rage, loneliness, and despair that result from unmet psychological needs. When these demands are not met, and the ensuing suffering is felt to be an intolerable state in the suicidal person, suicide is thought to be the best course of action. Psychological needs are what give a person their identity and drive them to live, and when those needs are not met, they can lead a person to decide to end their life.

We could characterize this as an unfulfilled need. Some examples of these psychological demands are achieving objectives like joining a buddy or group of people, attaining autonomy, opposing something, imposing oneself, and feeling accepted, understood, and comforted. Suicide is viewed as the most suitable solution for the suicidal person because of the agony that results from these requirements not being met and from this unacceptably painful state. There are psychological needs that define a person's life and personality, as well as psychological conditions that, when unmet, lead a person to decide to end their own life. From this vantage point, Shneidman believes that the best way to support suicidal people is to have a therapist who in some way attends to their unmet mental health needs by posing as a secular priest, an ombudsman, or an elderly woman who shops for them and depending on the idea of assistance. Continuous monitoring of suicide risk is crucial, taking into account warning indicators, such as alterations in behavior, particularly when accompanied by insomnia, and any expression of a desire to die. Individuals may have a sense of confinement and resort to unhealthy actions, such as consuming alcoholic beverages and utilizing psychotropic substances. Subjects contemplating suicide frequently exhibit behaviors such as organizing their personal matters and bestowing symbolic possessions, indicating a desire for someone else to assume responsibility for a cherished item, irrespective of its monetary worth. Orbach [28] examines the content analysis of pain narratives from suicidal patients and discusses several characteristics of the suicidal mindset. These include alterations in one's identity, instances of feeling disconnected from oneself, dissociative traits, a feeling of being devoid of value, emotional deprivation, and a decline in self-confidence. Moreover, the mind is frequently defined by the encounter of deprivation, such as occurrences that disrupt an individual's sense of ongoing identity and the loss of purpose in life. In addition, oxymoronic experiences involve intense contrasts in emotions, thoughts, and wants. These contradictions can include the simultaneous experience of living and dying and the conflicting feelings of grandiosity and shame. Moreover, the out-of-ordinary experience of pain highlights the inadequacy of conventional language in capturing these unique and individualistic feelings. Maltsberger [29] reported that intense despair is a mental emergency. Those patients who can escape it by turning to others for relief are fortunate. Some patients are able to access psychiatric therapy that alleviates their condition. Others end up falling back on drugs and alcohol to stem the anguish. However, many unfortunate patients may choose suicide as they cannot wait for this relief. Many desperate and anxious patients show how they are feeling with their facial expressions, body movements and behaviours, although many can seem quite calm and strangely calm. Potentially suicidal individuals must, therefore, be questioned about their emotional distress and whether and to what extent it is becoming intolerable. They should be asked to compare the severity of what they feel with other circumstances such as suicide attempts in the past. Those who appear calm may have already resolved their dilemma and have decided definitively to die by suicide. Having made the decision, others experience greater serenity, calmness and self-control before carrying out the lethal act. Still, others, to avoid having their suicide plans jeopardised, hide their desperation.

In this context, it is worth noting the model proposed by Maltsberger [29], which highlights the phenomena of ego failure (breakdown of the Self) in suicidal dynamics by proposing a model of suicidal collapse that involves four interconnected aspects. These aspects must not be understood as elements of a rigid sequence that follow one another according to an overdetermined order but rather as dynamic parts: one can see how patients can move back and forth from one aspect to another, observing a passage of level; some individuals show some parts more than others or even more than one aspect at the same time, but, regardless of the different individual characteristics, as suicide approaches, we observe how patients are more marked by the third and fourth parts of self-devolution (involution of the Self).

## Shifting to a new paradigm for suicide risk understanding

In contrast to the earlier consideration to limit the danger of suicide to the symptomatology of mental diseases, new understandings of the suicide phenomenon have led to the recognition that mental disorders do indeed contribute to suicide. However, a deeper comprehension of the suicidal mind is still required. Rather than labelling the suicidal person as having a mental disorder, medical professionals must be able to identify the psychological drama that is going on in the minds of people who may also be bipolar, depressed, or experiencing other psychiatric conditions. The majority of mentally ill do not die by suicide. Only when suicidal thoughts reside in a mentally ill person's brain and when suicidal emotions are so intolerable that suicide seems like the only option left to psychiatric sufferers. The majority of individuals with psychiatric conditions do not perish as a result of suicide. Psychiatric individuals exhibit suicidal tendencies when they experience intense negative feelings that are so unbearable that suicide seems the only viable choice. This occurs when the individual's mentally unstable brain harbors a suicidal mindset. It is a life-threatening behaviour “*combining features of a declaration of war with a petition for bankruptcy. If we limit our interpretation to the hostile phase, the suicidal act seems to be a punitive and destructive challenge to the world at large or to a specific set of significant people… when we stand at a distance from the turmoil of an individual who must fight or forfeit himself, our remoteness fosters the ‘the long view’ of war and business failure.* [30]. In other words, we must understand how the individual following a pathway of adversities and vulnerabilities became suicidal. Furthermore, the role of psychiatrists or clinicians should not be understood as being exclusive and decisive in preventive interventions in light of the bio-psycho-social etiology of the wish to die, with its profound social implications. This assumption is also important for the forensic perspectives of suicide [8].

Regarding suicide risk as only a symptom hinders the chance to research and comprehend suicide thoroughly. To understand, foresee, and manage suicide, it is essential to grasp the significance of suicidal thoughts and emotions for persons who experience them. As for mental pain as a pervasive experience, scholars reported that profoundly painful affective attacks are traumatic for the mind [31]. Such states often repeat early traumatic, painful conditions with the features of childhood neglect, with the crystallisation of self-destructive forms of self-abusive behaviour with failures in well-being, self-love, interpersonal relationships and harmony with reality, thereby making it very difficult to go on living [32]. Such individuals may develop a hopelessness perspective and consider suicide as an escape from suffering.

## Understanding unbearable mental pain means reasoning as phenomenological modalities

In meeting many suicidal individuals, I frequently experienced what the renowned Italian scholar Enrico Morselli referred to as moral pain [33], which encompasses negative emotions, such as shame, remorse, abandonment, dissatisfaction and lack of motivation, dysphoria, pessimism, inanition, and what Shneidman describes as psychache. When considering such matters, I perceive suicidal urges (thoughts and behaviors) from a phenomenological standpoint, akin to experiencing love rather than having an organ disease. There are striking examples of love as firsthand experience and knowledge. Love is known only through experience. It cannot be observed in any ‘objective’ way, but most of us recognise love as ‘real’, where love exists in the universe and is meaningful. Love, like most firsthand knowledge, is not generally discussed in suicide risk understanding. In other words, rather than looking for a given diagnosis, attention should be devoted to the essence of the individual and how emotions are experienced. Therefore, if the state of an individual is overwhelmingly beautiful, such as when one falls in love, then an overwhelmingly dreadful experience of mental pain invites suicidal intentions. It is a pervasive condition with psychic and somatic roots encompassing the individual. Suicides always happen when a person's personally defined psychological pain threshold is exceeded.

Discomfort is frequently felt in the hypochondrium and chest area. The mind incessantly seeks various avenues to alleviate anxiety, yet consistently fails to discover a secure refuge, ultimately becoming convinced that no remedy can provide solace. The injustice of being in such a situation often predominates.

The mind exhaustively explores every possibility to alleviate the tension, yet fails to discover a secure refuge and becomes sure that no solution would provide relief.

To differentiate suicidal ideation from psychiatric diagnosis, it is crucial to recognize that the factors contributing to the desire to end one's life form a distinct process, characterized by the unique reasoning of a distressed mind attempting to find a means to alleviate and overcome this anguish. The suffering that leads to suicide can be distinguished from the usual pain associated with depressive symptoms, as it arises from the individual's personality, unmet mental needs, and ego wounds, such as defeats, humiliation, and shame. Clinicians and patients may distinguish them. In the suicidal process, symptoms such as dysphoria, agitation, irritability, and anxiety are coupled with the fact that patients cannot stand the pain as if they were at a lowered threshold for suffering and see no possibilities other than death; due to the constriction of options left, the individual cannot see a way out and believes that ending their life is a solution [17].

Mental pain can be an important aspect to be assessed by a clinician. In the wake of the challenges posed by suicidal individuals, suicide risk assessment can be distressing for clinicians, which may favour the avoidance of in-depth exploration of suicidal thoughts and behaviour [34].

Research evaluating doctors' assessments of ideation and suicide risk revealed that doctors posed questions in a way that was likely to elicit a negative reaction, which was typically followed by a change of topic. This experience could be a factor in the high rate of people who claim not to have had suicide thoughts but then go on to commit themselves soon after [35].

Because of the ability to sustain a balanced risk formulation, developing a therapeutic and empathic connection between doctor and patient is essential to collaborative risk assessment and formulation. Building this kind of relationship requires a sincere and considerate relationship in which the therapist is aware of the impact a healing and compassionate human interaction can have on the patient's experience, involvement, and healing [34].

Clinicians are instructed to refrain from emphasizing prediction when formulating risk. This methodology disregards the assessment of risk based on categorized groups. It offers a framework for examining the biopsychosocial aspect of an individual's presentation, considering more than just their present emotions and thoughts. The objective of a formulation is to comprehensively comprehend the patient's experiences across their past, present, and future. This involves constructing a coherent narrative that elucidates the origins of the patient's current circumstances, behaviors, beliefs, thoughts, and actions and the impact these factors have had on their life. Furthermore, the formulation aims to identify potential avenues for future change or support. By comprehending a patient's history and current condition, we can more effectively strategize for symptom persistence, emergence, or reduction [36].

Building this relationship is based on a genuine and sympathetic connection, in which the clinician recognizes the influence that a therapeutic and compassionate human interaction can have on the patient's experience, engagement, and recovery. During the evaluation of recent and previous suicide actions, as well as present suicidal thoughts and actions, the clinician should compassionately, gradually, and truthfully assess suicidal ideation, motivation, intention, capability, and determination. This may involve explicitly inquiring the patient about their perspectives on suicide, as well as their reasons for wanting to continue living or considering death. Given how widespread suicidal thoughts is in the general population, Hawton et al. [34] stated that realizing this fact provides patients permission to tell whatever they might be embarrassed to share. The authors posited that a therapist's commitment to rapport-building enables the clinician to gently challenge the patient, highlighting any discrepancies between the patient's statements and the clinician's observations and nonverbal cues. For this, the clinician must be open to listening to the patient. The patient alone is the best authority on their own unique experiences. Moreover, a patient's adherence to subsequent therapy is determined by their initial meeting with a mental health practitioner. It takes an honest, nonjudgmental attitude to help patients reevaluate their conditions.

In this regard, two recent investigations focused on suicide risk, mental pain, childhood trauma, and the role of depressive symptomatology. These authors reported the role of mental pain in mediating between childhood traumatization and suicide risk status. The presence and severity of childhood trauma were associated with higher usual and worst mental pain in the last 15 days, and these were associated with increased suicide risk [37]. Moreover, patients who undergo intense mental pain and exhibit severe depressive symptoms, irrespective of their specific diagnosis, face an elevated risk of suicide [38].

It is strongly advised to investigate mental anguish in therapeutic settings to obtain more data for accurate evaluation of suicide risk and to enhance the empathetic comprehension of a patient's distress. Medical professionals should adopt a more comprehensive, cooperative, and inter-related approach with patients to gain insight into the mindset of those at risk of suicide [39–41]. The introduction of mental pain assessment is a way to address suicide risk; the clinician may stay in a strategic position to explore inner aspects of patients’ lives. When referring to suffering, the person allows themselves to be reached: the moment they can talk about their own emotional and cognitive sphere. It can report experiences related to the suicidal crisis, especially if the clinician is ready to explore its meaning together The interviewer should adopt an impartial and encouraging attitude. To achieve this, the clinician must possess a receptive attitude towards the patient, enabling them to actively listen and facilitate the patient's introspection and contemplation of their unique experiences. [42]. Shneidman emphasized the concept of anodyne therapy, which is a personalized approach aimed at relieving the specific psychological requirements of the patient that are causing frustration. Unfulfilled psychological demands are the underlying cause of suicide. Undoubtedly, the first objective of any therapy is to alleviate the patient's discomfort. He felt that the therapist should possess an understanding of the patient's psychological anguish and proceed with the “*mollification of that pain*”. It is important for clinicians to recognize that even small advancements can have life-saving effects as long as the patient acknowledges that pain can be endured to some extent. Shneidman [25] confidently asserted that this strategy enables clinicians to adjust and refine their methods by concentrating on the patient's unfulfilled psychological requests, which serve as a root cause of the patient's psychache. A proper understanding of the source of suffering should be put forward and dealt with, so that the wish to die because of that suffering is put aside.

In the state of unbearable mental pain as a whole private experience, patients perceive the uniqueness of their suffering and grandiosely assert that no one has had it as bad, but with a therapist’s efforts, words like “*unbearable and intolerable mean barely bearable and somehow tolerable, and that these insights can be incorporated into a scenario for long-term survival*” [25].

## Conclusions

There is a significant number of unmet needs for those at risk of suicide, and these needs are frequently dismissed as of secondary importance. There is also a great deal of psychological pain without suicide risk, which needs to be addressed and mollified among individuals either with a psychiatric diagnosis or just facing adverse circumstances.

Empirical evidence and current clinical practice indicate the necessity of developing a more comprehensive perspective of the mental state of those prone to suicide. Suicidologists, in line with Shneidman’s view consider suicide as a definitive resolution to a transitory issue characterized by overwhelming mental pain.

Every person is distinct, exhibiting their idiosyncratic manifestation of suicidal intentions. Nevertheless, most persons can attribute their suffering to particular unfulfilled requirements, enabling classification based on the absence of certain elements in their lives. Emblematically, modern psychiatry is reminded in the introduction of DSM-5 [43], that “*Diagnosis of a mental disorder should have clinical utility”* but *“the diagnosis of a mental disorder is not equivalent to a need for treatment. Need for treatment is a complex clinical decision that takes into consideration symptom severity, symptom salience (e.g., the presence of suicidal ideation), the patient’s distress (mental pain)*” and “*Clinicians may thus encounter individuals whose symptoms do not meet full criteria for a mental disorder but who demonstrate a clear need for treatment or care. The fact that some individuals do not show all symptoms indicative of a diagnosis should not be used to justify limiting their access to appropriate care*” (p. 20).

Contrary to being an unforeseen occurrence, suicidal behavior is distinguished by numerous indicators that frequently enable crucial therapeutic judgments, thereby saving the lives of those in distress. The task of suicide prevention is to sustain a culture diligently, both within clinical populations and the general public, that attends to the needs of individuals at risk of suicide, beginning with their fundamental unmet psychological needs. The Aeschii workgroup reported the goal for the clinician to be reaching, together with the patient, a shared understanding of the suicidal propensity [42]. This approach contrasts with the traditional medical approach, which places the clinician as an expert in identifying the causes of pathological behaviour and formulating a diagnosis. Stigmatisation and fear often provide reasons for empathic disconnection.

Moreover, even in cases where dedicated healthcare providers are open to acknowledging the patient's need, it is difficult to imagine the misery endured by these individuals.. For empathy to manifest, we must possess, within our own personal experiences and mental framework, certain points of reference that align with the patient's encounters with highly heightened levels of suicidal arousal or excitement [44] (and Maltsberger, 2010, personal communication). Establishing this relationship is founded on an authentic and compassionate connection, where the physician recognizes the impact that a therapeutic and empathetic human interaction can have on the patient's overall experience, involvement, and recovery. The is to employ a phenomenological methodology that focuses caregivers' attention on the subjective experience of psychological distress. While having an empathic understanding of the anguish experienced by a suicidal individual is not enough, it serves as the initial step in a potential process that could help prevent the individual from taking their own life. Pharmacological and non-pharmacological approaches are now available for the effective treatment of suicidal individuals, as well as for the treatment of psychiatric disorders that contribute to suicide risk. Such therapies can also be personalized if the inner mentalistic experience of suffering is explored.

The clinician's purpose should be to actively establish a mutual awareness of the patient's inclination towards suicide. Exploration of the source of sufferance other than mere diagnosis proves to be a strategic approach to reducing suicide risk. Modern psychiatrists may now have the chance to assess and manage suicide risk in their patients with an emphasis on mental pain. This feature often emerges from the combination of various instances. Collaborative and empathic risk assessment and formulation is, therefore, of crucial importance for changing the patients’ cognitions towards the wish to die.

## Data Availability

No data sets were generated or analysed during the current study.
